# Rotationally Adjustable Hyperthermia Applicators: A Computational Comparative Study of Circular and Linear Array Applicators

**DOI:** 10.3390/diagnostics12112677

**Published:** 2022-11-03

**Authors:** Gulsah Yildiz, Tuba Yilmaz, Ibrahim Akduman

**Affiliations:** 1 Department of Electronics and Communication Engineering, Istanbul Technical University, Istanbul 34467, Turkey; 2 Mitos Medical Technologies, Istanbul 34467, Turkey

**Keywords:** antenna excitation optimization, breast cancer, particle swarm optimization, specific absorption rate focus, microwave hyperthermia, hyperthermia treatment planning

## Abstract

Microwave breast hyperthermia (MH) aims to increase the temperature at the tumor location with minimal change in the healthy tissue. To this end, the specific absorption rate (SAR) inside the breast is optimized. The choice of the MH applicator design is important for a superior energy focus on the target. Although hyperthermia treatment planning (HTP) changes for every patient, the MH applicator is required to be effective for different breast models and tumor types. The linear applicator (LA) is one of the previously proposed applicator designs with linearly arranged antennas; however, it suffers from low focusing ability in certain breast regions due to its unsymmetrical geometrical features. In this paper, we propose to radially adjust the LA to obtain alternative excitation schemes without actually changing the applicator. Antipodal Vivaldi antennas were utilized, and the antenna excitations were optimized with particle swarm optimization (PSO). The comparison of the rotated and the fixed linear applicator, between 12-antenna circular and linear applicators, and finally, between a 24-antenna circular applicator are provided. Within the 12 rotation angles and two target locations that were analyzed, the 135° axially rotated linear applicator gave a 35% to 84% higher target-to-breast SAR ratio ($TBR_{S}$) and a 21% to 28% higher target-to-breast temperature ratio ($TBR_{T}$) than the fixed linear applicator. For the deep-seated target, the 135° rotated linear applicator had an 80% higher $TBR_{S}$ and a 59% higher $TBR_{T}$ than the 12-antenna circular applicator, while the results were comparable to the 24-antenna circular applicator.

## 1. Introduction

Microwave hyperthermia (MH) therapy aims to raise the tumor tissue temperature between 40–45° [1,2] using electromagnetic (EM) radiation in the microwave frequencies. MH has been used to increase the effectiveness of other cancer therapies such as radiotherapy or chemotherapy, as well as a sole treatment technique [3]. The steps of proper hyperthermia treatment planning (HTP) can be summarized as follows: After the patient is diagnosed with a malignant tumor, detailed imaging of the body part is obtained and the tissues are segmented [4,5]. Next, the accurate microwave dielectric and thermal properties of the tissues are assigned to each tissue type [6,7,8,9,10]. Then, the proper antenna excitations are applied for a controlled increase of the tumor temperature and hotspot prevention in the remaining healthy tissue, in other words the effective focusing of the specific absorption rate (SAR) on the target. Therefore, the optimization of the antenna excitation parameters (the phase and the power) is necessary for effective energy deposition over the target while maintaining low energy throughout the healthy tissue.

There are various techniques to obtain the proper antenna excitations. In previously reported hyperthermia studies, the time reversal (TR) technique was mainly used due to the general validity of its principle [11,12,13]. However, TR is prone to creating hotspots, especially in lossy media, where TR invariance is no longer valid. Particle swarm optimization (PSO) has been widely used in hyperthermia practices [14,15] due to its fast convergence; however, it suffers from the dependence of optimization parameters on random initial values, such that the result changes for each run. The same challenge arises also with differential evolution (DE) algorithms [16]. Other proposed approaches such as the Nelder–Mead simplex algorithm or convolutional neural networks do not depend on the initial value [17,18], but these are inferior to PSO and DE when compared in terms of the target-to-breast SAR ratio.

Traditionally, the MH applicator antenna excitation optimization is performed in two steps: first, the MH applicator design is chosen, and next, the optimization algorithm is applied for antenna excitation optimization. Therefore, the choice of the MH applicator is of great importance. Circular applicators (CAs) constitute the most common applications [19,20] in breast MH. The symmetry offered by the CA provides good coverage of the breast tissue. Linearly distributed applicator designs have also been proposed [18,21,22]; however, their application has not been appealing because they have low coverage due to the non-symmetrical geometry.

In this paper, the authors propose to physically rotate breast microwave hyperthermia applicators for better energy focusing on the target. Two designs of the MH applicators, linear and circular, each equipped with 12 antennas, were axially rotated by 15°, and each rotated applicator was analyzed and compared to one another. The antenna excitation parameter optimizations were conducted with PSO due to its wide use in the literature and fast convergence. Twelve rotations for the linear MH applicator and two rotations for the circular MH applicator were implemented. Lastly, the results of a circular applicator equipped with 24 antennas are given as a benchmark for the proposed system. In particular, the contributions of this paper can be summarized as follows:

- We propose to physically rotate microwave hyperthermia applicators about their center. This way, we employed 12 different designs for the linear MH applicator and two different designs for the circular MH applicator through rotational position re-adjustment.
- We showed that the proposed rotation feature increased the focusing capability of the linear MH applicator.
- We compared the results obtained from the two different MH applicator designs (linear and circular), which were also rotated.
- We compared the performance of the 24-antenna circular MH applicator with the 12-antenna circular, as well as 12-antenna linear MH applicators.
- We proposed the minimization of the average SAR at the healthy tissue.

The rest of the paper is divided into the following sections: Section 2 gives an overview of the hyperthermia problem. Section 3 gives the details of the methodology followed. Section 4 depicts the results of the proposed approach along with comparisons between the 12- and 24-antenna MH applicators, and the results are discussed in detail in Section 5. Lastly, the conclusions are drawn in Section 6.

## 2. Bio-Heat Equation

The microwave hyperthermia phenomenon is essentially the microwave heat transfer problem within biological tissues. Penne’s bio-heat equation governs this problem [23]:

(1) $$C_{p}\rho \frac{\partial T}{\partial t}=\nabla \cdot \left(K\nabla T\right)+A_{0}+\rho SAR-B\left(T-T_{b}\right)$$

where $C_{p}$ is specific heat capacity, ρ is the density, *K* is the thermal conductivity, *T* is the temperature, $T_{b}$ is the blood temperature, $A_{0}$ is the metabolic heat generation, and *B* is the capillary blood perfusion coefficient. These parameters are tissue-specific terms. The SAR is the specific absorption rate and proportional to the square of the electric field amplitude, $SAR=\frac{{0.5\sigma |E|}^{2}}{\rho }$ W/kg, where *E* is the electric field and σ (S/m) is the electrical conductivity. It was shown in [20] that the maxima of the SAR and the temperature are highly correlated, assuming *K* and *B* are constants and in a steady state, via Green’s function approach. Although a constant surface temperature, such as a cooling medium or water bolus, may alter this correlation by affecting the spatial differentiation of temperature due to the Dirichlet boundary condition, the correlation is still high to reach the desired target temperature by focusing the energy on the target within the SAR distribution.

## 3. Methods

### 3.1. Antenna Systems and Numerical Test Bed

**Figure 1:**
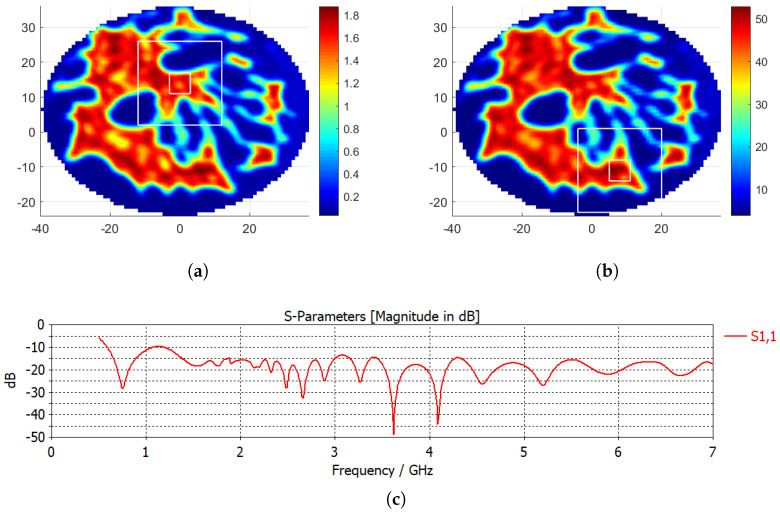
Dielectric properties of the central slice of the breast with tumor: (**a**) electrical conductivity (S/m), (**b**) relative permittivity (the axes are in mm). White squares on the conductivity graph indicate the target region (inner) and the healthy region (outer) boundaries for the target (0, 14, 0) mm, and white squares on the permittivity graph indicate the boundaries for the target (8, −11, 0) mm. (**c**) |S11| (dB) vs. frequency graph of the Vivaldi antenna.

In this study, it was assumed that the patient was scanned with magnetic resonance imaging (MRI) and that the dielectric properties of the breast were obtained from these MRI scans. In [24,25], the Debye parameters for glandular, fat, and skin tissues for various breast types were given. A healthy breast model, a heterogeneously dense breast with ID 062204 given in [24,25], was used in this study. The relative permittivity ($\epsilon_{r}$) and the electrical conductivity (σ) distributions at 2.45 GHz were calculated using the Debye parameters given in [25]. Figure 1a,b display the relative permittivity and electrical conductivity from the central slice of the breast phantom, respectively.

Two different hyperthermia applicator designs were analyzed in this work: linear and circular. Antipodal Vivaldi antennas, whose frequency range is given in Figure 1c, were employed as the EM sources with a 2.45 GHz operation frequency.

**Figure 2:**
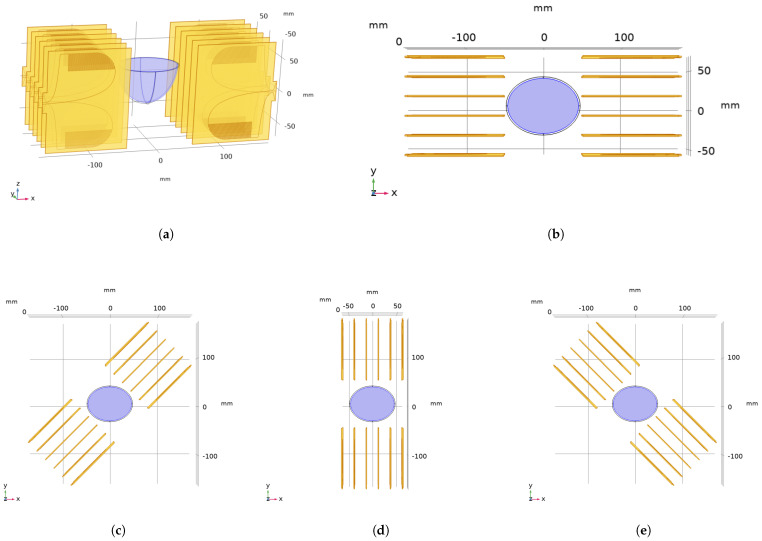
(**a**) Side view of the linear MH applicator with 12 antennas with a rotation angle of $\theta =0^{\circ }$. (**b**) Top view of the linear MH applicator with $\theta =0^{\circ }$. (**c**–**e**) Top views of the linear MH applicator with $\theta \;=\;45^{\circ },90^{\circ }$, and $135^{\circ }$, respectively.

Figure 2a shows the side view of the linear applicator (LA) with 12 antennas, where six antennas were placed linearly 2.5 cm apart on one side of the breast and the remaining six antennas were placed on the opposite side of the breast [21]. The polarization of the antennas should be carefully adjusted. The breast phantom and the applicator were located concentrically. The top view is depicted in Figure 2b. Then, the applicator was axially rotated with 15${}^{\circ }$ angular steps. Top views of the linear applicator with 45${}^{\circ }$, 90${}^{\circ }$, and 135${}^{\circ }$ rotation (θ) are shown in Figure 2c–e. The linear MH applicator with 12 antennas and x° rotation is referred to as $LA^{x}$ for convenience.

**Figure 3:**
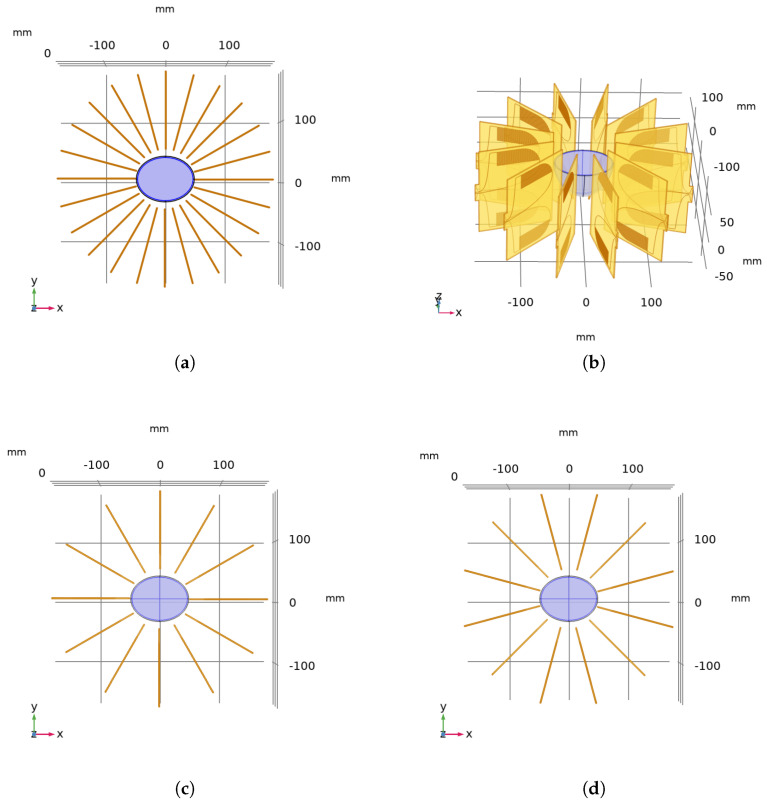
(**a**) Top view of the circular MH applicator with 24 antennas and $\theta =0^{\circ }$. (**b**) Side view of the circular MH applicator with 12 antennas and rotation angle $\theta =15^{\circ }$. (**c**,**d**) Top views of the circular MH applicator with 12 antennas and $\theta =0^{\circ }$ and $\theta \;=\;15^{\circ }$, respectively.

The circular applicator (CA) where 24 antennas were placed radially around the breast phantom is shown in Figure 3a, and a side view of the 12-antenna circular applicator is displayed in Figure 3b. Consecutive antennas had 30${}^{\circ }$ and 15${}^{\circ }$ angular separation for the 12- and 24-antenna CAs, respectively. The top view of the circular MH applicator with 12 antennas is depicted in Figure 3c. The applicator and the phantom are concentric, and the applicator was rotated axially by 15${}^{\circ }$; the geometry with $\theta =\;15^{\circ }$ is shown in Figure 3d. Note that the breast phantom was not moved during the rotation procedure. The circular MH applicator with 12 antennas and x° rotation is referred to as $CA^{x}$, and the circular MH applicator with 24 antennas is referred to as $CA_{24}$ for convenience.

### 3.2. Data Generation

Electromagnetic simulations were conducted with a finite-element-method (FEM)-based multi-physics simulation software, COMSOL Multiphysics. Antennas were excited with unitary excitation (1 Volt and 0 phase) individually, and the resulting electric field ($\vec{E}$) distributions at the central axial slice of the breast were obtained for every antenna and for every MH applicator rotation. Please see [18] for more details.

The total electric field vector inside the breast with N antenna excitations can be written as [26]:

(2) $${\vec{E}}_{tot}\left(r\right)=\sum_{i}^{N}a_{i}{\vec{E}}_{i}\left(r\right)e^{j\phi_{i}}$$

where $\vec{E_{i}}\left(r\right)$ is the electric field vector inside the breast when only the $i^{th}$ antenna is excited with unitary excitation and $a_{i}e^{j\phi_{i}}$ is the $i^{th}$ excitation coefficient with a $\phi_{i}$ phase difference and an amplitude of $a_{i}$. The phase of the first antenna was always kept as 0${}^{\circ }$ for a reference. The corresponding SAR is:

(3) $$SAR\left(r\right)=0.5\frac{\sigma \left(r\right)|{\vec{E}}_{tot}{\left(r\right)|}^{2}}{\rho (r)}\;W/\mathrm{kg}$$

at any location in the breast. To be able to compare different applicators and breast types and to increase the repeatability, the two-dimensional (2D) average operator is defined for the SAR as follows [3]:

(4) $$avSAR_{\Omega }=\frac{\sum_{\Omega }SAR}{Area\;of\;\Omega }\;W/kg\cdot m^{2}$$

where Ω is the surface of the 2D region. The main objective of the MH is to increase the SAR intensity at the target, while the healthy tissue SAR is kept low. To this end, the target-to-breast SAR ratio ($TBR_{S}$) and hotspot-to-target SAR quotient ($HTQ_{S}$) [3,18] metrics were used in this paper, and the formulas are given as follows:

(5) $$TBR_{S}=\frac{avSAR_{target}}{avSAR_{breast}}$$

(6) $$HTQ_{S}=\frac{avSAR_{hotspot}}{avSAR_{target}}$$

The area of Ω for the target region was chosen as a 6 mm × 6 mm square centered at the target point. The target region was then extracted, and the remaining breast was again divided into grids of 6 mm × 6 mm squares for hotspot determination. After the extraction of the target region, the region with the highest $avSAR_{grid}$ was assigned as the hotspot.

Two targets were analyzed for this paper. Target-I, which was a deeper-seated target, had the center position of (0, 14, 0) mm and target-II, a superficial target, had the center of (8, −11, 0) mm. The target (inner) and healthy (outer) region boundaries of target-I can be seen as the white squares in Figure 1a. Similarly, the white squares depicting the target and the healthy region boundaries of target II are given in Figure 1b. The healthy region is the area standing outside of the outer white square shown in Figure 1, and the corresponding average SAR at the healthy region can be calculated as follows:

(7) $$avSAR_{healthy}=\frac{\sum_{HealthyRegion}SAR}{\mathrm{Area}\;\mathrm{of}\;Healthy\;Region}\;W/kg\cdot m^{2}$$

Both targets were chosen embedded inside the glandular tissue, and the electrical properties of the target regions were the same as the background breast tissue. The dielectric properties of tumors are, in general, different from the healthy breast tissue and that, especially the higher conductivity of the tumors, helps SAR to be higher in the tumor [27]. In this paper, we chose more challenging cases such that the target had the dielectric properties of the background breast tissue and that the target was embedded inside the glandular tissue.

### 3.3. Optimization

Particle swarm optimization (PSO) with an inertia weight (iw-PSO) [28] was used to optimize the antenna excitation parameters, also known as the phase and the power of the individual antenna feed. It is a fast optimization technique, and it enables multi-parameter optimization and multi-objective cost functions. The parameters for iw-PSO were chosen as follows: c1=2.0, c2=2.0, and *w* starts from 1 and decreases with a damping constant of 0.99 in each iteration. A swarm size of 529 and an iteration number of 500 were used, and each case was run 50 times to determine the effect of the initial values, then the best-performing repetition was saved.

The cost function used within the SAR optimization is as follows:

(8) $$Cost=\frac{HTQ_{S}\cdot avSAR_{healthy}}{TBR_{S}}$$

where the healthy region is defined as the region $2\times r_{target}$ further away from the target point. The corresponding objective function can be written in the following form:

(9) $$\min_{\phi_{1,2,\ldots ,N},a_{1,2,\ldots ,N}}\frac{HTQ_{S}\cdot avSAR_{healthy}}{TBR_{S}}$$

### 3.4. Temperature Calculation

The obtained SAR distributions were fed into Penne’s equation given in Equation1 to obtain the temperature change in time. The calculation was performed with MATLAB with a finite difference time domain (FDTD) method and a Dirichlet boundary condition, such that the initial temperature and the boundary of the breast were kept at a fixed temperature of 37 °C. The thermal properties of the phantom were taken from [10]. Time was changed into 300 time steps with increments of two seconds, which is the maximum time step ensuring the convergence, completing 10 min of temperature increase. Spatial increments in both directions were taken as 1 mm. With a feedback loop, the SAR and $a_{1,2,\ldots ,N}$ were scaled until the average temperature at the desired target reached 43 °C in 10 min of application time.

To increase the sensitivity of the $TBR_{T}$ and $HTQ_{T}$ metrics in the table, the following change was made to the average operator of the temperature:

(10) $$avT_{\Omega }=\frac{\sum_{\Omega }\Delta T}{\mathrm{Area}\;\mathrm{of}\;\Omega }℃$$

where ΔT=(T−37°C). The area of Ω for the temperature target region was chosen as a 12 mm × 12 mm square centered at the target point. This is four-times larger than the SAR target region. Since the temperature distribution is a continuous function, there were no abrupt changes, and the target region was effective in a wider region. The target region was then extracted, and the remaining breast was again divided into grids of 12 mm × 12 mm squares for hotspot determination. After the extraction of the target region, the region with the highest $avT_{grid}$ was assigned as the hotspot.

The three metrics for the temperature, target-to-breast temperature ratio, hotspot-to-target temperature quotient, and the average temperature in the healthy region, are as follows:

(11) $$\begin{array}{cc} TBR_{T} & =\frac{avT_{target}}{avT_{breast}} \end{array}$$

(12) $$\begin{array}{cc} HTQ_{T} & =\frac{avT_{hotspot}}{avT_{target}} \end{array}$$

(13) $$\begin{array}{cc} avT_{healthy} & =\frac{\sum_{HealthyRegion}T}{\mathrm{Area}\;\mathrm{of}\;Healthy\;Region}℃ \end{array}$$

## 4. Results

First, the results of the 12 rotations of the linear MH applicator ($LA^{x}$) are given for target-I. The results were evaluated by means of both the SAR and temperature distributions with the use of the $TBR_{S}$, $HTQ_{S}$, $TBR_{T}$, and $HTQ_{T}$ metrics, as well as the average temperature at the healthy region.

Second, the results of the 12-antenna circular MH applicator are given for two rotations ($CA^{0}$ and $CA^{15}$) for target-I. Since the circular MH applicator inherently has a rotational structure, the comparison between the rotating linear and circular MH applicators is provided. Third, a comparison of the results of target-II is provided. Lastly, the results of the circular MH applicator equipped with 24 antennas are presented. This applicator has a 15° angle separation between its antennas and, therefore, can be viewed as the benchmark of the proposed system.

**Figure 4:**
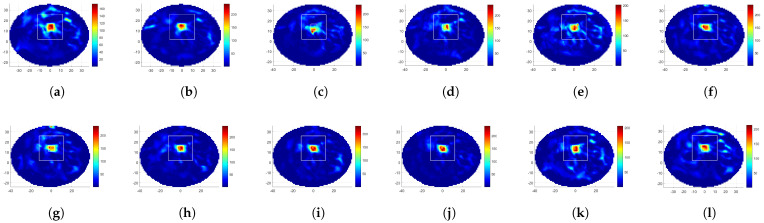
Results of focusing at target-I with the 12-antenna linear MH applicator: (**a**–**l**) SAR distribution (W/kg) for θ= 0°, 15°, 30°, 45°, 60°, 75°, 90°, 105°, 120°, 135°, 150°, and 165°. The white squares depict the target (inner) and healthy region (outer) boundaries used for the SAR and temperature metric calculations (the axes are in mm).

Figure 4 provides the SAR distributions for the 12 rotations of the linear MH applicator, where the SAR optimization was conducted for target-I. In a broad sense, the optimization procedure was successful as the maximum SAR level was at the target region. Looking at Table 1, the highest $TBR_{S}$ was obtained with $LA^{135}$, and the lowest $HTQ_{S}$ was obtained by $LA^{105}$ and $LA^{120}$. Comparing the result of rotated linear applicators with the fixed linear MH applicator ($LA^{0}$), applicators with rotations of 45°, 75°, 90°, 105°, 120°, 135°, and 150° were superior in the $TBR_{S}$ and $HTQ_{S}$ metrics.

**Figure 5:**
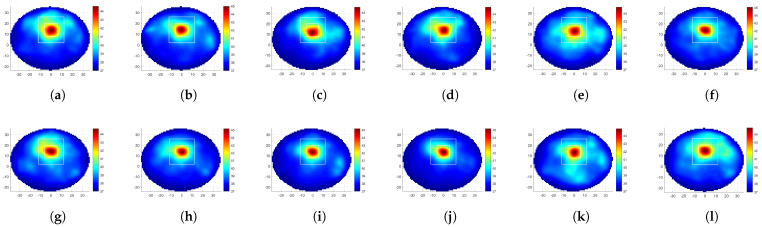
Results of focusing at target-I with the 12-antenna linear MH applicator: (**a**–**l**) temperature distribution (°C) for θ= 0°, 15°, 30°, 45°, 60°, 75°, 90°, 105°, 120°, 135°, 150°, and 165°. The white squares depict the target (inner) and healthy region (outer) boundaries used for the SAR and temperature metric calculations (the axes are in mm).

Figure 5 depicts the temperature distributions obtained with the SAR maps given in Figure 4. It can be observed that the maximum SAR and the maximum temperature positions were highly correlated. However, there was no direct matching between the SAR and the temperature distribution at the rest of the breast, and therefore, the analysis of the temperature was essential. The highest $TBR_{T}$ value was in line with the highest $TBR_{S}$ value and belonged to $LA^{135}$, while $LA^{120}$ held the lowest $HTQ_{T}$ value. $LA^{135}$ had an 84% higher $TBR_{S}$, 40% lower $HTQ_{S}$, 28% higher $TBR_{T}$, and 0.3 °C less average temperature at the healthy region compared to the fixed applicator.

**Figure 6:**
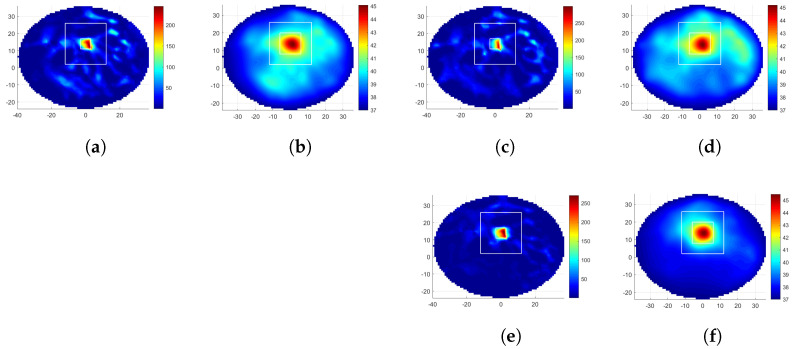
Results of focusing at target-I with the 12-antenna circular MH applicator: (**a**,**c**) SAR distribution (W/kg) for θ= 0°, 15°. (**b**,**d**) Temperature distribution (°C) for θ= 0°, 15°. Results of focusing at target-I with the 24-antenna circular MH applicator: (**e**) SAR distribution (W/kg). (**f**) Temperature distribution (°C). The white squares depict the target (inner) and healthy region (outer) boundaries used for the SAR and temperature metric calculations (the axes are in mm).

Figure 6a–c display the SAR distributions of the 12-antenna circular MH applicator with rotations θ= 0° and 15°, optimized to focus at target-I. Table 2 gives the corresponding $TBR_{S}$ and $HTQ_{S}$ metrics as 10.0, 0.33 for $CA^{0}$, and 7.9, 0.39 for $CA^{15}$, and $CA^{0}$ had better SAR-related metrics than $CA^{15}$ for target-I. Although the $CA^{0}$ metric was superior to $LA^{0}$, $LA^{15}$, $LA^{30}$, $LA^{60}$, and $LA^{165}$ in relation to $TBR_{S}$ and $HTQ_{S}$, they were inferior to the θ= 45°, 75°, 90°, 105°, 120°, 135°, and 150° rotated linear MH applicator results in relation to $TBR_{S}$, $TBR_{T}$, $HTQ_{T}$, and $avT_{healthy}$. Comparing $CA^{0}$ and $LA^{135}$, the latter one had a 0.8 °C less healthy tissue average temperature, a 59% higher $TBR_{T}$, and a 13% lower $HTQ_{T}$.

Table 3 gives the results of 12 rotations of the linear MH applicator that were optimized to focus at target-II. This target is closer to the surface. The highest $TBR_{S}$ was obtained with $LA^{135}$, while the lowest $HTQ_{S}$ was obtained with $LA^{15}$. The highest $TBR_{T}$ was in agreement with $TBR_{S}$, while the lowest $HTQ_{T}$ was obtained by $LA^{60}$. The least average healthy tissue temperature was also reached by $LA^{135}$. Applicators with rotations of 60°, 105°, 120°, 135°, and 165° had better metrics than the fixed applicator.

Table 2 shows the results for target-II for two rotated circular applicators with 12 antennas. $CA^{15}$ had higher $TBR_{S}$ and $TBR_{T}$ values. $CA^{15}$ had a lower $HTQ_{T}$ value than $CA^{0}$, and they had the same average healthy tissue temperature. Though these two rotated applicators had similar metrics, $CA^{15}$ was superior. Comparing $CA^{15}$ with $LA^{135}$, the latter had a 1% higher $TBR_{S}$, 4% higher $TBR_{T}$, and 28% higher $HTQ_{T}$, while $LA^{135}$ had a 0.1 °C less average healthy tissue temperature.

Figure 6e,f show the SAR and temperature distributions obtained with the 24-antenna circular MH applicator and optimized to focus at target-I. Table 2 gives the corresponding metrics. Looking at the results of the 24-antenna CA, $TBR_{S}$ was higher than the 12-antenna linear and circular applicators values, and it had the lowest $HTQ_{S}$. $CA_{24}$ had a low average healthy tissue temperature: 0.8 °C less than the 12-antenna CA and comparable to $LA^{135}$. $LA^{135}$ had a higher $TBR_{T}$ value than $CA_{24}$. The result of $CA_{24}$ for target-II, however, was superior to both 12-antenna applicators. It had the highest $TBR_{S}$, $TBR_{T}$, the lowest $HTQ_{T}$ value, and the lowest average healthy tissue temperature, which was only 0.3 °C higher than the nominal temperature of the body.

## 5. Discussion

The most-used applicator design in the literature is the circular array applicator. The main reason is that the circular shape of the applicator is compatible with the circular shape of the breast axial slice. Furthermore, the fixed linear applicator does not have symmetrical radiation, and the circular applicator, on the contrary, is symmetrical; thus, the radiated EM energy is expected to behave similarly for different target locations. This difference between the linear and the circular applicators would result in favoring the circular applicator. The comparison of the already published works is given within the provided results, referred to as “the fixed geometry’. Comparing these fixed circular and linear arrays of antennas by means of their performance on the energy deposition onto the target, the circular array applicator was shown to be the superior one. This result supports the fact that the circular array is the state-of-the-art. This work, however, demonstrated that, with the help of radial re-positioning, the linear array applicator could be comparable to or more beneficial than the circular applicator with the same number of antennas. Not only does the linear applicator benefit from this feature, but also, the rotating circular applicator provided better results than the fixed one for target-II. However, the improvement was not as distinct as in the linear applicator, due to the inherent symmetry that CA holds. The improvement would be more apparent if there were fewer antennas in the applicator. With eight antennas, for example, the rotation within CA could be 30°, contrary to the 15° that the 12-antenna CA has.

For target-II, the LA rotation giving the best metrics was 135°. This was an expected result since the position of the antennas is closer to the target than the fixed applicator, as can be seen in Figure 2e, though the exact rotation would be closer to 120°. With target-II, $LA^{90}$ was expected to give the best metrics by means of proximity to the target; however, $LA^{135}$ gave better metrics. This can be explained by the homogeneity of the radiation path. The variation of the dielectric properties of the breast was lower at the 135° angle, and the radiation path had fewer fluctuations in comparison to the path of the 90° angle. The best practice would be to analyze all the possibilities for a well-designed HTP.

The rotating 12-antenna linear MH applicator gave superior results to the 12-antenna circular applicator, and more investigation on the usage of the proposed system should be conducted. The rotation feature provides a variety of focusing options, from which the physician can choose to continue with, without actually changing the applicator itself. A basic MH system would require an antenna system, amplifiers (preferably adjustable), phase shifters, a power generator, and a control system. The complexity of the setup can be increased for a clinical prototype. The antennas are generally mounted to a casing that fixes the individual antennas. The proposed rotation feature can be easily added in two separate ways. One of the ways is to use a circular rail, on which the antenna casing can slide, and the rotation can be adjusted by the user. Another way is that the casing can be connected to a motor, where the rotation can be performed with the use of a control system. Both configurations enable the rotation of the whole system without altering the relative positioning of the individual antennas. However, the choice of rotation degree should be determined before the application via the EM simulations of the proposed system in a patient-specific scheme. To do so, first, the degree of rotation, as well as the antenna excitation parameters should be optimized. Then, the setup should be adjusted accordingly, either by hand or by a motor, and finally, the hyperthermia application can be conducted in the envisioned practical setting. It should be noted that this paper did not provide optimization on the power requirement of the applicators, and a comparison based on that would be misleading.

The temperature inside the target region changed between 40.6 °C and 45 °C, while the average temperature was the same for all the cases because the SAR distributions were scaled so that the average temperature at the target region was 43 °C. Hence, the average temperature of the healthy tissue becomes an important metric to investigate. Lower temperature values for the healthy region are preferable as hyperthermia therapy requires the lowest effect outside of the target area.

Having a 15° separation between its antennas, the 24-antenna CA can be seen as a benchmark for the proposed systems. The 24-antenna circular applicator provided better results by a large margin for target-II; however, this was not the case for target-I. In practice, using a high number of antennas would require more equipment for the RF back-end such as phase shifters, amplifiers, power dividers, and other microwave components, which increases the cost of the end product.

## 6. Conclusions

In this paper, we proposed to use the advantage of the rotation of an MH applicator to obtain different excitation schemes without actually changing the applicator itself. We were able to analyze 12 and 2 different excitation schemes for linear and circular applicator designs, respectively. The results showed that the quality of the focus obtained with different rotations was diverse. The quality mostly depended on the location of the target region inside the breast and the surrounding tissue properties, and the analysis of each rotation before the treatment planning provides important information and, possibly, a better HTP. The 135° axially rotated linear applicator gave 35–84% higher $TBR_{S}$ and 21–28% higher $TBR_{T}$ values than the fixed linear applicator for the implemented breast phantom and the targets. The rotated 12-antenna linear MH applicator presented superior or comparable results to the rotated 12-antenna circular applicator, as well, in most of the given metrics. Within the analyzed targets, for target-I, the 135° rotated linear applicator had 80% higher $TBR_{S}$ and 59% higher $TBR_{T}$ values than the 12-antenna circular applicator, while the results were very close for the 24-antenna circular applicator. For target-II, the results of the 12-antenna linear and circular applicators were comparable to each other, and the 24-antenna circular applicator had 26% higher $TBR_{S}$ and 26% higher $TBR_{T}$ values than the 135° rotated 12-antenna linear applicator. However, using 24 antennas would have twice the product cost of 12-antenna applicators. The analysis of the power consumption of the applicators, different antenna numbers, and different rotation angle studies need to be further examined. 

## Figures and Tables

**Table 1:** Focusing results of the linear MH applicator at target-I with changing the antenna number and rotation angle.

Target	Target-I											
θ	**0°**	**15°**	**30°**	**45°**	**60°**	**75°**	**90°**	**105°**	**120°**	**135°**	**150°**	**165°**
${\mathrm{TBR}}_{S}$	9.8	9.2	7.8	11.5	8.5	11.9	10.2	15.0	15.1	18.0	11.0	9.1
${\mathrm{HTQ}}_{S}$	0.50	0.36	0.64	0.41	0.40	0.33	0.47	0.26	0.27	0.30	0.31	0.36
${\mathrm{TBR}}_{T}$	3.6	3.6	3.8	3.6	3.1	3.7	3.5	3.8	4.1	4.6	3.1	3.1
${\mathrm{HTQ}}_{T}$	0.55	0.55	0.54	0.63	0.58	0.50	0.63	0.59	0.49	0.54	0.56	0.60
${\mathrm{avT}}_{\mathrm{healthy}}$(°C)	38.1	38.2	38.0	38.1	38.4	38.1	38.2	38.1	38.0	37.8	38.4	38.4

**Table 2:** Focusing results at two targets of the circular MH applicators with 12 and 24 antennas and changing the rotation angle.

Applicator	12-Antenna CA				24-Antenna CA	
**Target**	**Target-I**		**Target-II**		**Target-I**	**Target-II**
θ	**0°**	**15°**	**0°**	**15°**	**0°**	**0°**
${\mathrm{TBR}}_{S}$	10.0	7.9	15.0	16.5	18.1	21.2
${\mathrm{HTQ}}_{S}$	0.33	0.39	0.60	0.65	0.18	0.53
${\mathrm{TBR}}_{T}$	2.9	2.7	5.3	5.6	4.5	7.2
${\mathrm{HTQ}}_{T}$	0.62	0.65	0.53	0.43	0.48	0.45
${\mathrm{avT}}_{\mathrm{healthy}}\left({}^{\circ}C\right)$	38.6	38.8	37.6	37.6	37.8	37.3

**Table 3:** Focusing results of the linear MH applicator at target-II with changing the antenna number and rotation angle.

Target	Target-II											
θ	**0°**	**15°**	**30°**	**45°**	**60°**	**75°**	**90°**	**105°**	**120°**	**135°**	**150°**	**165°**
${\mathrm{TBR}}_{S}$	12.4	9.9	11.7	13.7	13.3	11.7	12.5	14.4	13.1	16.7	14.1	15.3
${\mathrm{HTQ}}_{S}$	0.80	0.40	0.59	0.47	0.71	0.51	0.83	0.65	0.69	0.59	0.67	0.62
${\mathrm{TBR}}_{T}$	4.8	3.6	3.9	4.3	4.9	4.1	4.3	5.2	5.4	5.8	4.8	5.4
${\mathrm{HTQ}}_{T}$	0.53	0.60	0.52	0.50	0.48	0.49	0.56	0.49	0.55	0.55	0.68	0.56
${\mathrm{avT}}_{\mathrm{healthy}}$ (°C)	37.7	38.1	38.1	37.9	37.7	38.0	37.9	37.7	37.6	37.5	37.8	37.6

## Data Availability

Not applicable.

## Abbreviations

The following abbreviations are used in this manuscript:

MH	Microwave hyperthermia
SAR	Specific absorption rate
CNN	Convolutional neural network
EM	Electromagnetic
TR	Time reversal
iw-PSO	Inertia-weighted particle swarm optimization
HTP	Hyperthermia treatment planning
FDTD	Finite difference time domain
ISM	Industrial, Scientific, and Medical
MRI	Magnetic resonance imaging
TBR	Target-to-breast ratio
HTQ	Hotspot-to-target quotient
CA	Circular applicator
LA	Linear applicator
TC	Tumor coverage
